# Examining the Formalin Fixation Method for Maintaining High RNA Quality in Surgical Lung Specimens

**DOI:** 10.1111/1759-7714.70005

**Published:** 2025-01-23

**Authors:** Takashi Teishikata, Manabu Itoh, Yusuke Okamoto, Naofumi Miyahara, Chiho Nakashima, Koichiro Takahashi, Masafumi Hiratsuka, Keita Kai, Keiji Kamohara

**Affiliations:** ^1^ Department of Thoracic and Cardiovascular Surgery Faculty of Medicine, Saga University Saga Japan; ^2^ Division of Hematology, Respiratory Medicine and Oncology, Department of Internal Medicine Faculty of Medicine, Saga University Saga Japan; ^3^ Department of Pathology Faculty of Medicine, Saga University Saga Japan

**Keywords:** lung cancer, multiplex gene‐panel testing, RNA preservation

## Abstract

**Background:**

Multiplex genetic testing is recommended when treating nonsmall cell lung cancer. A certain percentage of test failures in RNA assays owing to poor surgical specimen quality have been documented, and fixation failure is a possible cause. At our institution, sheet‐like fixation is performed to reduce fixation time. This study aimed to compare the quality of RNA from resected lung cancer specimens following different fixation methods.

**Methods:**

Sheet‐like fixation specimens (*n* = 15), conventional fixation specimens of the same resected lungs (*n* = 15), and other lung cancer specimens collected for conventional fixation and subjected to multiplex gene‐panel testing (*n* = 22) were retrospectively examined. RNA was extracted from each specimen. RNA quality and quantity were compared, and the success rate of multiplex gene‐panel testing was determined.

**Results:**

The DV_200_ value was significantly higher in RNA extracted from sheet‐like fixation samples (median 47.5%, interquartile range [IQR]:40.3–51.5) compared with RNA extracted from conventionally fixed samples or conventionally fixed samples of other patient specimens (median 21%, IQR:5.3–29.8 and median 16.3%, IQR:9.5–27.1, respectively). No significant difference was observed in nucleic acid concentration. The multiplex genetic analysis success rate was 95% with conventional methods (one failure); however, it was 100% with the sheet‐like fixation method.

**Conclusion:**

Sheet‐like fixation preserved RNA extracted from lung cancer specimens, resulting in lesser degradation than with conventional fixation.

## Graphical abstract

This study aimed to assess the quality of RNA obtained from sheet‐like fixation lung cancer samples. The quality and concentration of RNA in histopathological samples obtained using sheet‐like and conventional fixation methods, in addition to the success rate of genetic testing, were compared. The DV200 value of RNA extracted from sheet‐like fixation samples was significantly higher than that extracted from conventional fixation samples, and there was no failure in multiplex gene‐panel testing.

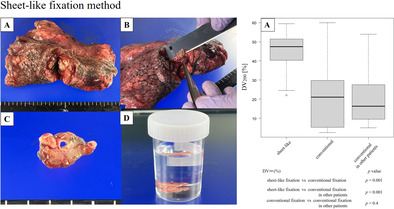

## 1 Introduction

Many driver gene abnormalities have been identified in nonsmall cell lung cancer (NSCLC), resulting in advancements in the development and clinical adoption of targeted drugs [1]. In NSCLC, oncogenic driver mutations are commonly found in genes such as epidermal growth factor receptor (*EGFR*), erb‐b2 receptor tyrosine kinase 2 (*HER2*), v‐raf murine sarcoma viral oncogene homolog B1 (*BRAF*), Kirsten rat sarcoma viral oncogene homolog (*KRAS*), anaplastic lymphoma kinase (*ALK*), ROS proto‐oncogene 1 (*ROS1*), RET proto‐oncogene (*RET*), and hepatocyte growth factor receptor (*MET*) [2]. Various guidelines recommend identifying driver gene alterations before systemic therapy [3, 4]. Gene‐panel analysis using next‐generation sequencing (NGS), multiplex reverse transcriptomes, or real‐time polymerase chain reaction (RT‐PCR) should be performed to simultaneously search for multiple driver gene alterations [5, 6].

Formalin fixation and paraffin embedding (FFPE) is the preferred method for preserving clinical tumor specimens, and the requirement for high‐quality FFPE DNA extracts has increased in recent years [7]. In general, to avoid unsuccessful analysis, the samples submitted for multiplex gene‐panel testing should be of sufficient quantity, requiring higher nucleic acid quality than that required for conventional single‐gene tests [8]. Although surgical specimens are easier to obtain than biopsy specimens, proper specimen processing is important for successful multiplex genetic testing analysis. The poor quality of specimens remains a pressing issue, especially in RNA assays using surgical specimens, where test failures are still observed. The larger the specimen, the more likely the ribonuclease is not completely inactivated, thereby increasing the failure rate of RNA analysis [9]. Therefore, at our hospital, resected lung tissue is quickly divided into sections and fixed in sheet form, known as sheet‐like fixation.

This study aimed to assess the quality of RNA obtained from sheet‐like fixation of lung cancer samples. We compared the quality and concentration of RNA from histopathological samples obtained using sheet‐like and conventional fixation methods and determined the success rate of genetic testing.

## 2 Materials and Methods

### 2.1 Samples

All samples (*n* = 22) submitted for genetic testing using conventional fixation methods and 15 cases fixed in the sheet form from patients treated for lung cancers at our hospital from January 2023 to February 2024 were included. Samples for sheet‐like fixation and conventional fixation (maximum split surface) were included in the sheet‐fixed case group (*n* = 15, respectively).

For each case, we collected information regarding age, sex, surgical procedure, lung cancer histopathology, pathological invasion size, and gene alteration analysis results of the primary lung cancer tissue.

Multiplex gene‐panel testing was performed using either the Oncomine Dx Target Test (ODxTT) (Ion Torrent PGM Dx Sequencer; Thermo Fisher Scientific, Waltham, MA, USA) or the Amoy Dx Pan Lung Cancer PCR panel (AmoyDx) (Amoy Diagnostics Co. Ltd., Xiamen, China) or the Lung Cancer Compact Panel (compact panel; DNA Chip, Tokyo, Japan). The specimens used for RNA extraction were the sections used for genetic testing in the conventional fixation group and sheet‐like fixed sections or the largest segment of conventionally fixed tumor in the sheet‐like fixation group. The duration from tissue fixation to RNA extraction for all samples was consistently < 14 months.

This study was approved by the Ethics Committee of Saga University Faculty of Medicine (approval number: 2024‐02‐R‐01). Informed consent was obtained using an opt‐out method.

### 2.2 Conventional fixation method

Conventional fixation methods were performed following The Japanese Society of Pathology Guidelines on the Handling of Pathological Tissue Samples for Genomic Medicine [10]. Resected lung cancer specimens were immediately fixed with 10% neutral buffered formalin (within 1 h). Formalin was injected into the bronchi or through the pleura until the lung was dilated, allowing the formalin to permeate. The fixation time was 48 h.

### 2.3 Sheet‐like fixation method

**FIGURE 1:**
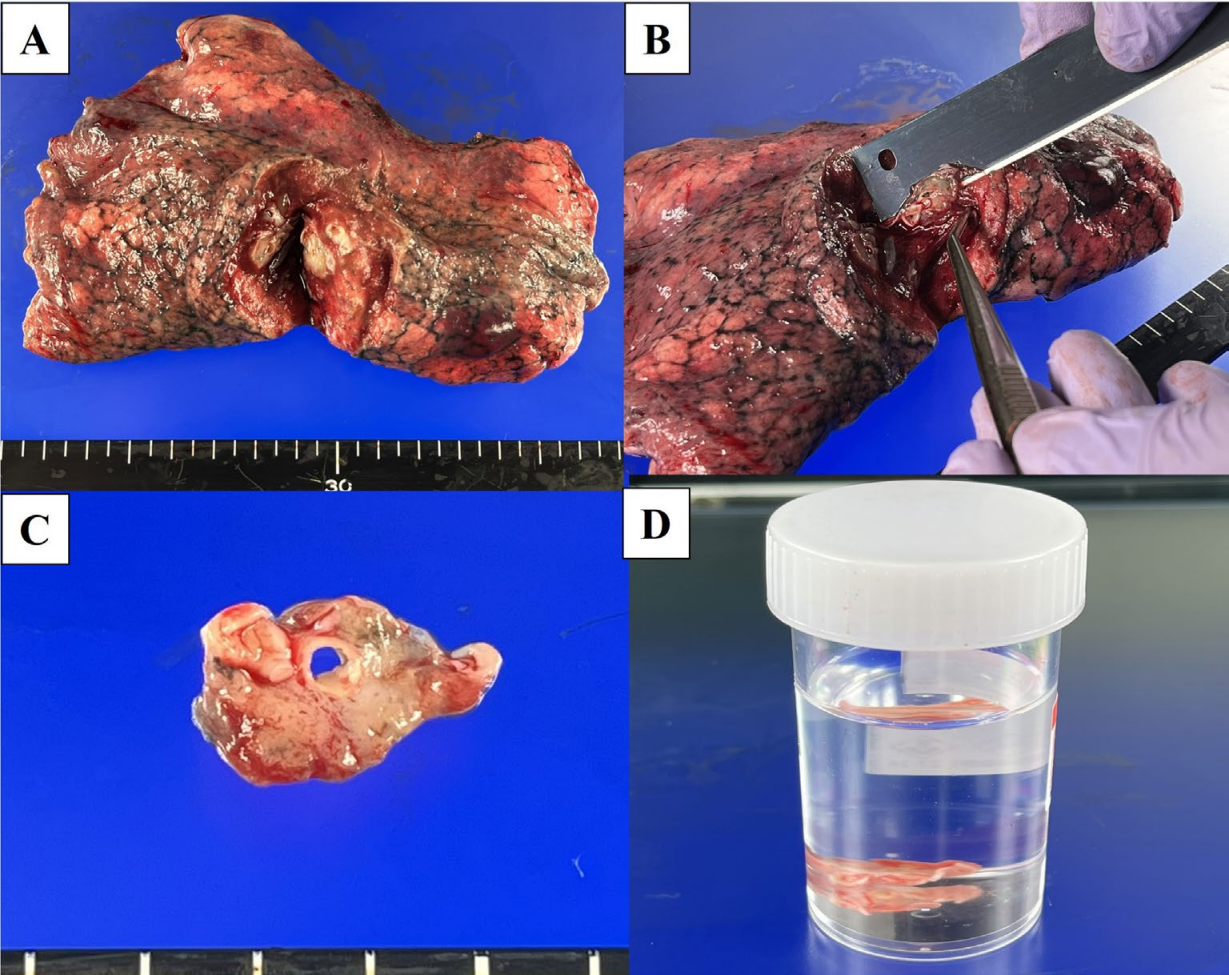
Sheet‐like fixation method (freshly resected specimen from the right lower lobe) for the subtotal cut near the center of the tumor (A). Thin sections measuring approximately 15 mm × 15 mm and 1‐mm thick (B, C). Fixation in 10% neutral buffered formalin vial (D).

The sheet‐like fixation technique is illustrated in Figure 1. Immediately following resection, an incision was made at the center of the tumor, avoiding the pleural indentation. A 15 mm × 15 mm sized, 1–2‐mm thick sheet‐like sample was obtained and fixed in a 10% neutral buffered formalin vial for genetic testing.

The incision was lightly sutured to the pleura, and 10% neutral buffered formalin was injected with a syringe through the bronchi or lung parenchyma, as in the conventional fixation method, until the lung was maximally expanded. The fixation time was 48 h. Collected specimens were assessed for suitability for genetic testing using hematoxylin and eosin staining and, if suitable, were subjected to multiplex gene‐panel testing. If there was severe necrosis or fibrosis on the slice surface and the tumor size or tumor content was small, the slices were considered unsuitable.

### 2.4 RNA extraction

Total RNA was purified from three FFPE tissue sections of 10‐μm thickness. In the sheet‐like fixation group, sheet‐like fixed samples of the tumor were used, while in the conventional fixation samples, the largest split surface of the tumor was used; in the conventional fixation samples of the other patient groups, the same sections used for genetic testing were used. For RNA isolation, the tissues were further processed using the automated Maxwell RSC (Promega, Mannheim, Germany) platform and the commercially available Maxwell RSC RNA FFPE kit (AS1440, Promega). RNA was isolated according to the manufacturer's instructions.

### 2.5 Determination of RNA quantity and quality

The total RNA concentration of each sample was determined using a NanoDrop 2000 (Thermo Fisher Scientific, Wilmington, DE, USA) spectrophotometer. The size distribution of the RNA was examined through capillary electrophoresis. We used the RNA 6000 Piko kit (Agilent Technologies Inc., Santa Clara, CA, Product no. 5067‐1513) and a chip and analyzed it using an Agilent 2100 Bioanalyzer equipped with Expert 2100 software (Agilent Technologies Inc., Santa Clara, CA, USA), according to the manufacturer's instructions. The DV_200_ and RNA integrity number (RIN) values were automatically calculated against internal standards using Agilent 2100 Expert software version B.02.08. The RIN was generated using an entire electrophoretic trace and algorithm, and the values ranged from 1 (lowest quality) to 10 (highest quality). DV_200_ represents the percentage of RNA fragments > 200 nucleotides. All experiments were conducted in duplicate, and results were compared based on the mean values.

### 2.6 Statistical Analysis

Categorical variables are expressed as numbers (percentages) and were compared using Fisher's exact test or Pearson's chi‐square test, as appropriate. Continuous variables are expressed as medians with interquartile ranges (IQRs) and were compared using the Mann–Whitney *U* test. The threshold for significance was set at *p* value less than 0.05. All statistical analyses were performed using R version 3.5.3 (The R Foundation for Statistical Computing, Vienna, Austria) with EZR version 1.38 (Saitama Medical Center, Jichi Medical University, Saitama, Japan).

## 3 Results

### 3.1 Patient Characteristics

Thirty‐seven patients were enrolled in this study, and their clinical characteristics are shown in Table 1. Fifteen and 22 patients were enrolled in the sheet‐like fixation and conventional fixation groups, respectively. The average ages were 76 and 72 years, respectively, showing significant differences. There were no significant differences in terms of sex, operative procedure, histopathology, size of pathologic invasion, or type of genetic testing.

**TABLE 1:** Patient characteristics.

Variables		Sheet‐like fixation (*n* = 15)	Conventional fixation in other patiants (*n* = 22)	*p*
Age, (median, IQR)		76 (72.5–80)	72 (65.3–74.8)	0.007
Sex, *n*	Male	8	12	1
	Female	7	10	
Operative procedure, *n*	Lobectomy	14	15	0.204
	Segmentectomy	1	5	
	Partial resection	0	2	
Tumor histology, *n*	Adenocarcinoma	11	14	0.883
	Squamous cell carcinoma	3	5	
	Others	1	3	
Pathological tumor size, mm, (median, IQR)		34.8 (29.8–54.9)	35.2 (26.8–59.6)	0.781
Multiplex gene panel testing type, *n*	Oncomine DxTT	9	9	0.01
	AmoyDx	1	13	
	Compact panel	1	0	

### 3.2 RNA quality and quantification

**FIGURE 2:**
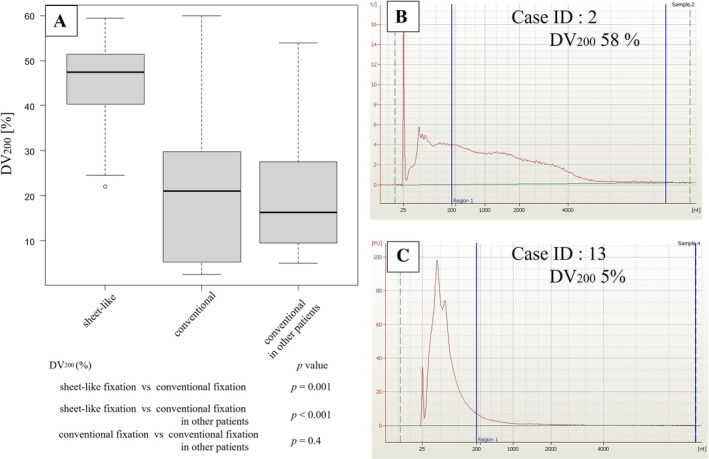
Comparison of RNA quality with each method: DV_200_ (A). Representative DV_200_ of two cases. The case of conventional fixation method (B) and sheet‐like fixation (C).

The DV_200_ values of RNA determined using the bioanalyzer are shown in Figure 2A and Table 2. The median DV_200_ for RNA was 47.5% for the sheet fixation method, compared to 21% for the conventional fixation method for the same resected specimens. The median DV_200_ of RNA was 16.3% for conventional fixation of different resected lung cancer specimens. The DV_200_ values were significantly better for sheet fixation than for conventional fixation. There was no significant difference in value between the two groups where samples were assessed by the conventional method.

**TABLE 2:** Comparison among the three groups.

Variables	Sheet‐like fixation (*n* = 15)	Conventional fixation (*n* = 15)	Conventional fixation in other patient (*n* = 22)	*p* ^a^	*p* ^b^
DV200, % (median, IQR)	47.5 (40.3–51.5)	21 (5.3–29.8)	16.3 (9.5–27.1)	0.001	< 0.001
Concentration, ng/μL (median, IQR)	17.2 (11.5–25.3)	18.6 (13.9–31.7)	16.2 (12.5–23.7)	0.539	0.877
RIN, (median, IQR)	2.3 (2.2–2.4)	2.4 (2.3–2.5)	2.4 (2.3–2.5)	0.065	0.004
Period from sampling until RNA extraction, months (median, IQR)	11 (10–12)	11 (10–12)	11 (10.2–12.8)	1	0.422

^a^ Statistical differences between Sheet‐like fixation and conventional fixation groups.

^b^ Statistical differences between Sheet‐like fixation and conventional fixation in other patient groups.

The median RNA concentrations for sheet‐like fixation, conventional fixation, and conventional fixation in other patients were 17.2, 18.6, and 16.2 ng/μL, respectively. There were no significant differences in RNA concentrations among group. In contrast, the median RIN values of the sheet‐like fixation, conventional fixation, and conventional fixation in other patients were 2.3, 2.4, and 2.4, respectively, which were low; however, RIN values were significantly lower with the sheet‐like fixation. No significant difference was observed between each group in terms of the period from sampling to RNA extraction; the three groups were analyzed at a median of 11 months.

### 3.3 Multiplex gene‐panel testing

The multiplex gene‐panel analysis results are shown in Table 3. Sheet‐like fixed samples were successfully analyzed in all cases. The success rate of conventional fixation samples was 95%, and only one sample had insufficient nucleic acid content; the DV_200_ of this sample was 10% and the RNA concentration was 5.8 ng/μL (Table 4). The driver mutation detection frequency following genomic testing was 50% for conventional fixation and 36% for sheet‐like fixation. Four sheet‐like fixation samples were not subjected to multiplex panel testing.

**TABLE 3:** Comparison of the results of multiplex gene panel testing between the two groups.

Variables	Sheet‐like fixation (*n* = 11)	Conventional fixationin other patiants (*n* = 22)	*p*
Success rate of gene multi test, %	100%	95%	
Success	11	21	1
Failure	0	1	
Total	11	22	
Positive	4 (36%)	11 (50%)	0.712
*EGFR* ex19del	2	1	
*EGFR* L858R	1	5	
*EGFR* G715A	0	1	
*KRAS* others	0	2	
*HER*2 ex20ins	0	1	
*MET* ex14skipping	1	1	
Negative	7 (64%)	11 (50%)	
Period from sampling until multiplex gene panel testing, days (median, IQR)	35 (25–45)	37 (28–43)	1

**TABLE 4:** One failure case.

DV200, %	10
Concentration, ng/μL	5.8
RIN	2.25
Period from sampling until multiplex gene panel testing, days	35
Multiplex gene panel testing type	Amoy Dx

## 4 Discussion

In this study, we evaluated the quality, quantitation, and success rate of multiplex gene‐panel testing of RNA extracted from resected lung cancer specimens using two different fixation methods. The DV_200_ value of the RNA extracted from sheet‐like fixation samples was significantly higher than that of RNA extracted from conventional fixation samples, and there was no failure in multiplex gene‐panel testing. Molecular therapy targeting driver mutations is a clinical standard; therefore, the mutational status of driver mutations must be identified to determine therapeutic options for patients. The number of identified target genes continues to increase, and single‐gene tests no longer cover all target genes; therefore, multiplex gene‐panel testing is recommended in the molecular testing guidelines [3, 4].

FFPE specimens are used for genetic testing; however, attention must be paid to the degradation of nucleic acids recovered from FFPE specimens. Nucleic acids derived from FFPE specimens are highly degraded and denatured, which may reduce the accuracy of analysis results or become insufficient for use in genetic analysis. Although molecular techniques are currently optimized for analyzing formalin‐fixed tissue with fragmented DNA and RNA, delayed fixation results in the lysis of DNA and, especially, RNA by tissue D(‐R)NAses [11, 12]. The diffusion rate of neutral buffered formalin in the lung tissue may not be the same as that in other solid organs, as the lung parenchyma entrapped in the remaining air in the partially collapsed lung may have an obstructive effect on fixative diffusion. The edge of the specimen was immediately fixed in the first hour, whereas the center area was likely fixed in the last hour. This implies that time‐dependent impaired tumor fixation is present centrally in every resected specimen [13, 14, 15]. Poor fixation of the tumor center may occur in fixed lung resection specimens. In contrast, the sheet‐like fixation method involves taking a central part of the tumor and fixing it in a sheet to reduce the fixation time and ensure firm fixation. Other reports similarly collected samples of 10 mm × 10 mm for genetic testing and reported good results in gene‐panel testing [16, 17]. In these previous reports, comparisons were made using different tumor samples, with no detailed examination of how fixation methods within the same tumor affect RNA quality [16, 17]. In our study, we focused on the different methods of fixing the same surgical specimen. We showed that fixing the specimen in sheet form significantly improved the quality of the RNA. The thickness was set at 1–2 mm for better specimen handling and 15 mm × 15 mm to obtain a wide range of tissue from even heterogeneous tumors or tumor tissues showing necrosis. These results strongly indicate the usefulness of our sheet‐like fixation method as a new approach to improve RNA quality, which has been challenging to enhance with conventional methods.

Several RNA quality indices have been developed, including the RIN and DV_200_. The DV_200_ was developed by Agilent in 2014 as a tool to accurately assess the quality of RNA samples and has been used as an RNA quality assessment standard, even in the protocol published by Illumina. The DV_200_ is a useful RNA quality index for NGS analyses using degraded RNA samples, such as those extracted from FFPE samples [18]. The RIN has been widely used as an indicator of RNA quality in NGS, microarrays, and qPCR. The DV_200_ is more suitable than RIN for the quantification of RNA because it can be applied to evaluate RNA extracted from fresh or frozen samples and samples with low RIN values, such as RNA extracted from FFPE samples [19, 20, 21]. In our study, the DV_200_ was significantly better using the sheet fixation method. In contrast, RIN values were better with the conventional method; however, as both arms had RIN values < 2.5, it is difficult to interpret the role of the RIN.

Typically, a longer storage time increases degradation and reduces the lengths of sequence fragments that can be amplified [22]. The DV_200_ for sheet fixation was significantly better than that in the other groups but less than 50. This may be due to the fact that the median storage period for all samples in this study was approximately 1 year. The fixation time was set at 48 h. The advantage of sheet fixation is that the fixation time can be shortened by using thin slices, and a fixation time of 48 h may be too long. Shortening the fixation time can improve quality.

Evaluation of the one case of failure by conventional methods revealed evidence of RNA fragmentation and low levels of accommodative concentrations of nucleic acids. This underscores the potential limitations of the conventional method in handling certain sample conditions and further supports the robustness of the sheet‐like fixation method.

This study has some limitations. First, it was a retrospective analysis performed at a single institution, and the sample size was relatively small. In particular, the number of genetic tests was small, and a large number of cases was required. Conversely, the specimen processing procedures were relatively uniform because the study was performed at a single institution. Second, the time period between sampling and multiplex gene‐panel testing for all samples was within 2 months, which does not reflect the poor quality of the RNA extracted at this time. This study assessed the quality of the RNA at approximately 1‐year after fixation, at which point samples may have been of lower quality than that during the multiplex gene‐panel testing. Further studies are required to collect a large number of specimens to help reduce sampling errors. Third, we only examined the quality of RNA and did not examine the quality of DNA. Since gene multi‐tests are performed on DNA and RNA, it is necessary to evaluate the quality of DNA as well.

## 5 Conclusions

Sheet‐like fixation showed higher DV_200_ values than the conventional fixation method. This method may increase the success rate of genetic testing in institutions that use 10% neutral buffered formalin to fix surgical specimens.

## Author Contributions

**Takashi Teishikata:** writing – reviewing and editing, writing – original draft preparation, methodology, conceptualization. **Manabu Itoh:** writing – reviewing and editing, methodology, conceptualization. **Yusuke Okamoto:** data curation. **Naofumi Miyahara:** data curation, writing – reviewing and editing. **Chiho Nakashima:** data curation, writing – reviewing and editing. **Koichiro Takahashi:** writing – reviewing and editing. **Masafumi Hiratsuka:** data curation, writing – reviewing and editing. **Keita Kai:** writing – reviewing and editing. **Keiji Kamohara:** conceptualization, writing – reviewing and editing. All authors critically reviewed and revised the manuscript draft and approved the final version for submission.

## Conflicts of Interest

The authors declare no conflicts of interest.

## Supporting information


**Data S1.** Supporting Information.

## Data Availability

The datasets generated during and/or analyzed during the current study are available within the article and supplementary data files. The raw data are available from the corresponding author on reasonable request.
